# A Psychometric Study of the Prosocial Behavior Scale: Differential Item Functioning by Gender

**DOI:** 10.3390/bs13030259

**Published:** 2023-03-15

**Authors:** Sara Martínez-Gregorio, José M. Tomás, Amparo Oliver

**Affiliations:** Department of Methodology for the Behavioral Sciences, Faculty of Psychology and Speech Therapy, University of Valencia, Av. Blasco Ibáñez 21, 46010 Valencia, Spain

**Keywords:** differential item functioning, psychological capital, validation, prosociality, adolescence, psychometric properties

## Abstract

Some scales co-exist in the literature to measure prosocial behavior in adolescents. Gender differences in prosocial behavior have been a controversial topic of research. To strengthen future research in the area, the psychometric properties of the most used instruments must be guaranteed, especially its gender non-bias. Our study provides psychometric evidence for the Prosocial Behavior Scale in a sample of adolescents, exploring: (a) its factor structure; (b) reliability; (c) gender-related differential item functioning (DIF); (d) nomological validity. A sample of 512 high school students (mean age = 13.62 (SD = 1.34), 51.6% females) participated in the research. Confirmatory Factor Analysis (CFA) was used to test the factor structure of the scale, which adequately fitted the data (χ^2^ (35) = 152.224, *p* < 0.001, Comparative Fit Index (CFI) = 0.905, Root Mean Squared Error of Approximation (RMSEA) = 0.085 90%CI [0.072–0.099], Standardized Root Mean Squared Residual (SRMR) = 0.079). Reliability results were good (α = 0.74, ω = 0.74). Regarding the DIF, five items presented some gender-related bias, generally benefiting females. However, the DIF impact could be considered negligible. Correlations with the subdimensions of the psychological capital offered evidence of the nomological validity of the scale. In conclusion, the scale presented adequate psychometric properties that support its ability to effectively assess prosocial behavior and gender differences in the prosocial behavior samples of adolescents. Additionally, the results obtained imply that gender differences in the manifestations of prosocial behavior require measurements that can fairly sample behaviors characteristic of each gender.

## 1. Introduction

Prosocial behavior refers to voluntary actions conducted to benefit others [1]. Although this behavioral tendency would be positive along the lifespan, it may be crucial in some early stages as adolescence. Previous empirical research supports the idea that prosocial behavior promotes positive academic outcomes [2], psychosocial adjustment [3,4], and well-being [5,6].

Different self-reported scales for assessing prosocial behavior in adolescents co-exist in the literature. Table 1 includes the scales most widely used internationally. Some of them approach the construct from multidimensional perspectives, whereas others present a unidimensional structure. The Objective Measure of Prosocial Reasoning (PROM) method [7] includes stories that invoke a conflict between adolescent needs, wants, and desires and those of others. The respondents are asked to decide what the main character of the story should do, showing their prosocial reasoning. Another multidimensional worldly spread instrument is the Prosocial Tendencies Measure—Revised (PTM-R) [8]. This is the adapted version for adolescents of the Prosocial Tendencies Measure (PTM) [9]. Not being that common, the Teenage Inventory of Social Skills (TISS) [10] measures prosocial behaviors such as cooperation or altruism in one of their two sub-scales. Complementary, the other one assesses asocial behaviors such as aggression or social anxiety.

Broad instruments for adolescent adjustment can also include a dimension for prosociality. That is the case for the Strengths and Difficulties Questionnaire (SDQ) [11]. The SDQ is a screening tool for assessing psychosocial adjustment in children and adolescents. Despite not being prosocial-specific, it presents a five-item subdimension that measures prosocial behavior. The use of this dimension as a prosociality scale is interesting because it has been included in the National Health Survey in Europe [12]; therefore, a large quantity of data is available. The SDQ has alternative versions wherein the rater is a teacher or the parents [12,13]. Although our work is focused on self-reported prosociality scales, there are other alternatives for assessing prosocial behavior with external raters, such as the prosocial with peers subscale in the Child Behavior Scale (CBS) [14] or the parent-report Brief Adolescent Prosocial Perceptions Scale (BAPPS-P) [15]. The latter has a self-reported alternative, the BAPPS-S. However, to date, it has been scarcely used.

The last scale widely used to assess prosocial behavior is the Prosocial Behavior Scale (PBS) [16], which is different from the Adult Prosocial Behavior Scale [17]. PBS is a 10-item unidimensional scale. Originally, it included 15 items; however, five of them are control items that are not taken into consideration for the total prosocial behavior score. It has been mainly used in a Spanish-speaking context, where we can find many articles using it in the last 20 years [18,19,20,21,22,23,24,25,26,27,28,29,30,31,32]. Although being widely used, a proper psychometric study for the Spanish adaptation has never been carried out. Some papers refer to Del Barrio et al. [33] to justify the adequate psychometric properties of the instrument. However, it is a widespread mislead. Del Barrio et al. [33] studied the psychometric properties of the Aggression and Emotional Instability scales from Caprara and Pastorelli [16], but not the psychometric properties of the PBS. Through previously cited research, we know that its reliability results range between 0.60 and 0.85 [18,19,20,21,22,23,24,25,26,27,28,29,30,31,32]. However, only two previous studies have reported evidence about its factor structure [19,26]. Carlo et al. [19] found problems with one item and reported the Confirmatory Factor Analysis (CFA) results of a reduced version with nine items. Consequently, as far as our concern, Malonda et al. [26] were the first authors to report a satisfactory adjustment of the unidimensional structure.

This lack of a properly complete and more updated psychometric study of the PBS affects specifically the validity related evidence. For example, some relevant aspects such as the gender-related differential item functioning remain understudied. By gender-related differential item functioning, we refer to the fact that the item score depends not just on the prosocial behavior of the respondent, as it would also vary considering their gender. Gender differences, while studying global prosocial behavior, consistently benefit females [34]. Some authors have argued that, rather than a matter of grade differences in the actual prosocial behavior, there are perhaps differences in how each individual, females and males, acts prosocially [35]. Eagly [36] concluded that females present more prosocial behavior related to communal attributes, whereas males’ prosocial behavior may be more agentic. Consequently, it has been criticized that the items of prosocial behavior scales may typically reflect gender-role stereotypes, being more prevalent on the female-type of prosocial behavior [37]. Xiao et al. [38] conducted a meta-analysis of gender differences in adolescents using the PTM-R. They found that gender differences were higher in those dimensions considered “gender-type prosocial behavior”. Females report higher levels of altruistic, compliant, and emotional prosocial behavior, whereas males present higher levels of public prosocial behavior [38]. Similarly, Nielson et al. [37] noticed that, on prosocial behaviors oriented to peers, females presented higher emotional support, inclusion, and sharing.

**Table 1:** Instruments for assessing prosocial behavior in adolescents.

Scale	Original Authors	N Items	Dimensions	Psychometric Studies	Recent Applications
Objective Measure of Prosocial Reasoning (PROM)	Carlo et al. [7]	56 items	Hedonistic, needs-oriented, approval, stereotypic, and internalized	English [7]; Spanish [27,39]; Chinese [40]; Portuguese [41]	[42,43]
Prosocial Behavior Scale (PBS)	Caprara and Pastorelli [16]	10 items	Unidimensional	Italian [16]; Spanish [26]	[20,32]
Prosocial Tendencies Measure—Revised (PTM-R)	Carlo et al. [8]	25 items	Altruistic, compliant, emotional, dire, public and anonymous	English [8]; German [44]; Lithuanian [41]; Chinese [45]; Portuguese [46]; Spanish [30]	[38,47]
Strengths and Difficulties Questionnaire (SDQ)—Prosocial dimension	Goodman [11]	5 items	Unidimensional	English [11]; Chinese [48]; Dutch [49,50]; German [51]; Norwegian [52] Spanish [12,53,54]; French [55]; Swedish [51,56]; Cypriot Greek [51]; Italian [51]	[57,58]
Teenage Inventory of Social Skills (TISS)	Inderbitzen and Foster [10]	40 items	Prosocial behavior and asocial behavior	English [10]; Spanish [59]	[60]

The complexity of the measurement of gender differences in prosocial behavior requires robust psychometric studies that guarantee adequate item functioning. These efforts have been previously carried out when studying the gender invariance of the SDQ [12,53], the PTM-R [18,30], and the PROM [39]. All of them were reported in a multigroup CFA analysis conducted to examine invariance across gender. However, not all of them achieved a scalar gender invariance. Ortuño-Sierra et al. [53] did not test scalar invariance, and only tested the equivalences of the structure and factor loadings, that is, metric invariance. Carlo et al. [39] did not find support for the scalar gender invariance of the PROM, finding differences in five-item intercepts.

Although previous research has used the PBS to explore gender differences in prosocial behavior [18,24,61], its gender-related differential item functioning has never been analyzed. Research with other scales of prosociality suggests that this aspect is crucial to effectively measure prosociality in both genders, not being affected by a gendered vision of prosociality. Consequently, given the importance of the PBS in a Spanish context, a proper psychometric study to guarantee its reliability and validity in its Spanish form is required. Therefore, our study aims to evaluate the psychometric properties in its Spanish adaptation. The study has four objectives: (a) to verify its one-dimensionality; (b) to test its reliability; (c) to explore its gender-related differential item functioning; (d) to collect evidence of its nomological validity. The following hypotheses are derived from these objectives:

**Hypothesis** **1** **(H1).** *The PBS has a unidimensional structure.*

**Hypothesis** **2** **(H2).** *The reliability results of the scale in Spanish adolescents are adequate.*

**Hypothesis** **3** **(H3).** *The items of the PBS do not present gender-related differential functioning.*

**Hypothesis** **4** **(H4).** *The results of the PBS are positively related to the psychological capital of the students, as evidence of nomological validity* [62,63].

## 2. Materials and Methods

### 2.1. Participants

The sample was composed of 512 high school students from Valencia, Spain. The mean age was 13.62 (SD = 1.34). Of the participants, 51.6% (*n* = 264) were females and 45.5% (*n* = 233) were males. Nine students (1.8%) self-identified themselves as “other” and six did not answer the question. Regarding their high schools, 57.22% (*n* = 293) attended public ones, whereas 26.37% (*n* = 135) attend private or semi-private ones. Most of the respondents were studying compulsory secondary education: 132 (25.8%) in their first course, 83 (16.2%) in their second course, 190 (37.1%) in their third course, and 72 (14.1%) in the fourth course. Twenty-five students were in post-compulsory secondary education, baccalaureate: 21 (14.1%) were in their first year and 4 (0.8%) were in the second and final year. The remaining 2% (*n* = 10) did not declare which course they were attending.

### 2.2. Procedure

The sampling for the study was performed at the convenience of the educational centers that were supportive of the research. The only inclusion criteria were that the students were in secondary education and that their families agreed to their participation; no exclusion criteria were considered. The survey and procedure met the American Psychological Association (APA) Ethical Standards for human research and were approved by the Research Ethics Committee of the University of Valencia as well as the Educational Government of Valencia (UV-INV_ETICA-1218680). The research team contacted the high school education authorities to present the research project and ask for their authorization to send the information to the families. In those high schools willing to participate, the families and students were informed about the research, and they signed informed consent. Participation in the survey was anonymous, voluntary, and non-rewarded.

Once informed consent was received, students answered the survey in class. The estimated duration for the entire survey was 20–25 min; however, there was no time constraint for any of the participants. Students who did not wish to participate remained in the classroom studying on their own until the rest of the classmates finished the questionnaire. As data were recruited in class, the lack of response from the participants was negligible (less than 1%).

### 2.3. Instruments

Alongside the socio-demographic questions (age, gender, course, and high school), two questionnaires were administered for the current research:

- Prosocial Behavior Scale (PBS) [16]. Although this scale includes 15 items, just 10 of them ask for prosocial behavior. The remaining five items are control items that do not contribute to the total prosocial behavior score (items PB3, PB6, PB8, PB11, and PB14). This research only analyzes those items measuring prosocial behavior. However, the items are named through the manuscript, retaining their original numeration from 1 to 15. The item content is presented in Table 2. In this study, the items were administered in its Spanish form. The answer format is a 3-point Likert scale (1—never, 2—sometimes, and 3—often).

- Psychological Capital Questionnaire (PCQ-12) [64]. This questionnaire was recently adapted and validated for Spanish adolescent samples by Tomás et al. [65]. It measures the four subdimensions of psychological capital in the educational context: self-efficacy (three items), hope (four items), resilience (three items), and optimism (two items). An example item is “I usually take stressful things at school in stride”. Following Tomás et al.’s [65] adaptation, we used a 5-point Likert response format, ranging from 1—strongly disagree to 5—strongly agree. The four-factor structure with a second-order factor fits adequately our data: χ^2^(50) = 269.181, *p* < 0.001, Comparative Fit Index (CFI) = 0.936, Root Mean Squared Error of Approximation (RMSEA) = 0.095 90%CI [0.084–0.106], and Standardized Root Mean Squared Residual (SRMR) = 0.052. The reliability results for our sample administration were for self-efficacy (α = 0.81, Ω = 0.85), hope (α = 0.76, Ω = 0.81), resilience (α = 0.37, Ω = 0.38), and optimism (α = 0.61; Ω = 0.67).

### 2.4. Data Analysis

The psychometric properties of the PBS were assessed following four steps: (1) factor structure, (2) descriptive statistics and reliability, (3) differential item functioning, and (4) nomological validity.

The factor structure was assessed using Confirmatory Factor Analysis (CFA) carried out in Mplus 8.7 [66]. Due to the categorical nature of the response scale, corrected Weighted Least Square Mean and Variance (WLSMV) was used as the estimation method. Some indices were considered to evaluate the adequacy of the unidimensional structure of the data [67], including (1) the chi-square test; (2) the CFI (adequate if above 0.9); (3) the RMSEA (adequate if below 0.08); (4) the SRMR (adequate if below 0.08). Full Information Maximum Likelihood methods were used for missing data, as they are reliable for missing at random (MAR) and missing completely at random (MCAR) missingness [68].

Some descriptive statistics for the items were calculated with IBM SPSS Statistics 26, i.e., means, standard deviations, skewness, kurtosis, inter-items correlations, and corrected item-total correlations. Reliability was assessed via Cronbach’s alpha and McDonald’s omega. Both indicators are considered adequate if their value is above 0.7 [69].

Next, the Differential Item Functioning (DIF) was evaluated in R [70], using the lordif package [71]. Based on the Graded Response Model, the lordif package tests DIF, estimating three nested proportional-odds logistic regression models. Model 1 expresses the cumulative probabilities that the true item response falls into if a specific response category is higher based on one’s level in the trait (in this case, the prosocial behavior). Model 2 adds the effect of the grouping variable (gender) on this probability, or uniform DIF. Model 3 includes the interaction of the trait and the grouping variable to assess non-uniform DIF. From this framework [72], the contrast of the fit of the models, in terms of the likelihood ratio test (χ^2^), allows us to detect different kinds of DIF. Uniform DIF is tested by comparing likelihood values for Models 1 and 2, whereas non-uniform DIF is tested by comparing Models 2 and 3. Additionally, a statistically significant result in the chi-square test between Models 1 and 3 could be interpreted as a “total DIF effect” [71]. Some authors also propose that the magnitude of DIF could be measured through changes in the *pseudo*-R^2^ statistics. We employed the McFadden *pseudo*-R square. Zumbo [73] set some cut-off criteria to interpret them, being results under 0.13 and 0.26 and thus considered negligible and moderate, respectively. Results above 0.26 were considered large. The DIF analysis was followed with some visual analyses of the plots the lordif package offers for items with DIF and the individual-level DIF impact.

Lastly, nomological validity was assessed through Pearson’s correlations between psychological capital subdimensions and the total score of prosocial behavior (PB). Based on previous literature, the psychological capital could be consider as a precursor of prosocial behavior [62,63]. Consequently, positive correlations between both constructs indicated the nomological validity of the PBS. This analysis was performed in IBM SPSS Statistics 26. To complement DIF analyses, a new aggregate measure of prosocial behavior (PB *), without including items with DIF, was computed and correlated with the psychological capital subdimensions and PB. Correlation comparisons were conducted between PB and PB * correlations with the psychosocial capital dimensions to assess DIF’s impact on nomological validity.

## 3. Results

### 3.1. Factorial Structure

**Figure 1:**
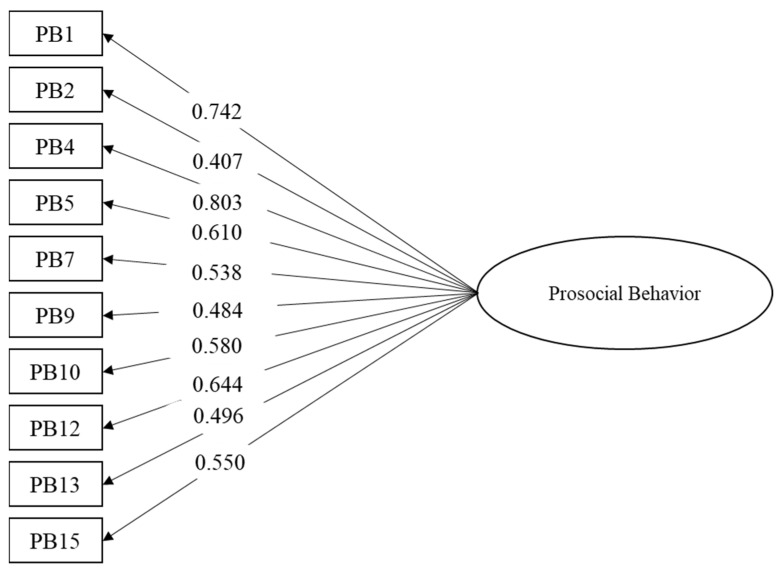
Factorial structure. Note: all the factor loadings are statistically significant *p* < 0.001.

A CFA was specified and tested to verify the theoretical unidimensional structure. The model fit adequately with the data: χ^2^ (35) = 152.224, *p* < 0.001, CFI = 0.905, RMSEA = 0.085 90%CI [0.072–0.099], SRMR = 0.079. Figure 1 shows the standardized factor loadings of the items. All of them are well above 0.3, ranging between 0.407 and 0.803.

### 3.2. Descriptive Statistics and Internal Consistency

Table 3 includes the descriptive statistics (mean, standard deviation, skewness, kurtosis, inter-item correlation, and corrected item-total correlation) of the items. Considering that the Likert scale ranges between 1 and 3, all the item means are above the mid-point. The item-total correlations are all above 0.3. However, item 2 presents the lowest item-total correlations with non-statistically significant inter-item correlations in items 4, 5, and 9. Item 2 had the lowest factor loading, as shown in Figure 1. The reliability results for the scale are good, with a Cronbach’s alpha of 0.74 and a McDonald’s omega of 0.74.

### 3.3. Gender-Related Differential Item Functioning

**Figure 2:**
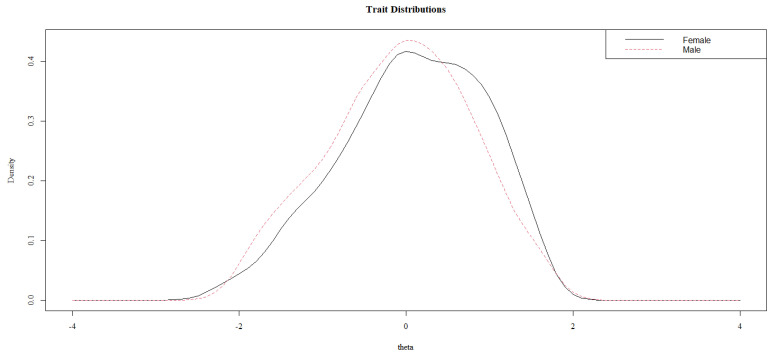
Theta distributions by gender.

Figure 2 shows the theta distribution for females and males. Although males show a slightly lower level of prosocial behavior, there is a broad overlap in the distributions. Table 4 presents the results for the χ^2^ tests between Model 1 and Model 2, Model 1 and Model 3, and Model 2 and Model 3 for all items. The statistically significant difference between Model 1 and Model 2 indicates the presence of a uniform DIF, whereas the statistically significant difference between Model 2 and Model 3 denotes a non-uniform DIF in the item. The statistically significant chi-square test between Model 1 and 3 may be interpreted as an overall test of the “total DIF effect”. The results in Table 4 indicate that items PB1, PB4, PB12, PB13, and PB15 present DIF. All of them present uniform DIF; however, items PB1 and PB12 also present non-uniform DIF. The non-uniform DIF in these items indicates that the DIF varies depending on the prosocial behavior level. However, considering the *pseudo*R^2^ changes, the DIF in all the items may be considered negligible [73].

**Figure 3:**
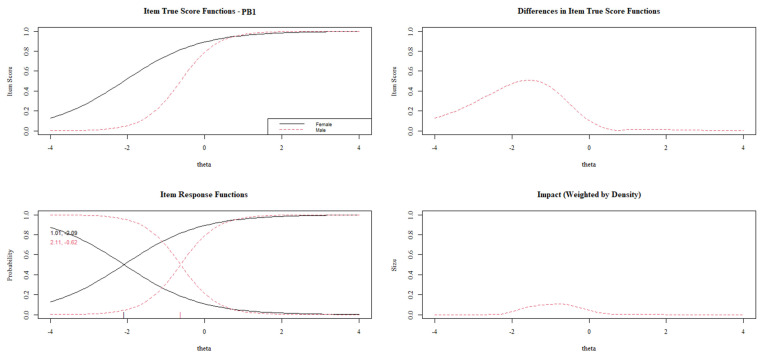
DIF plots for item PB1.

**Figure 4:**
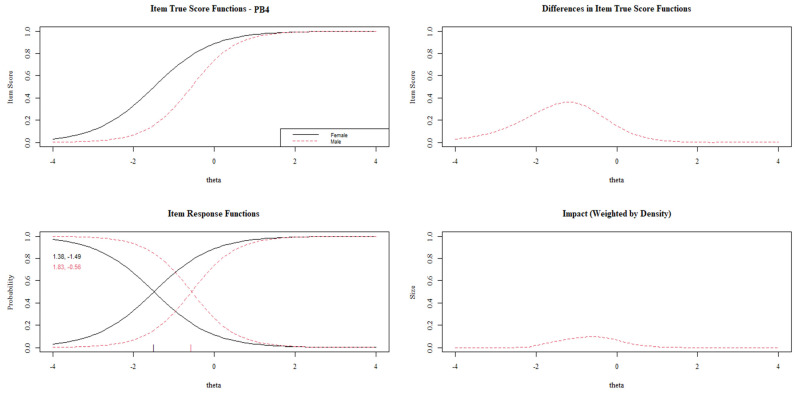
DIF plots for item PB4.

**Figure 5:**
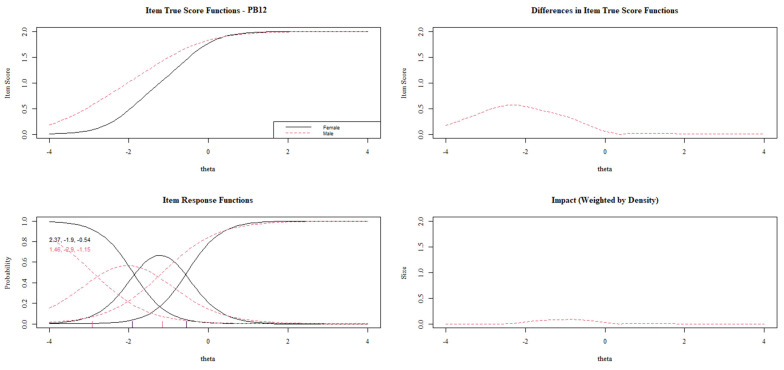
DIF plots for items PB12.

**Figure 6:**
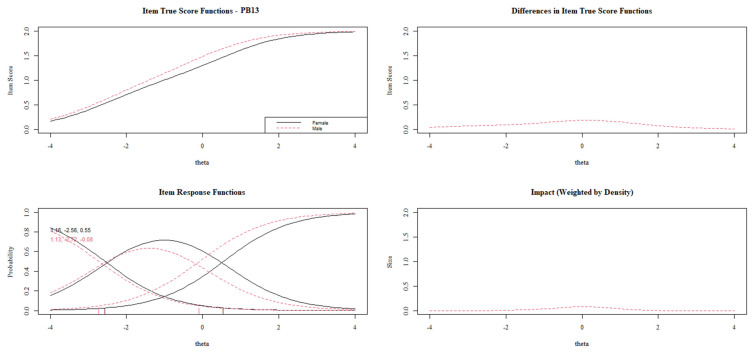
DIF plots for item PB13.

**Figure 7:**
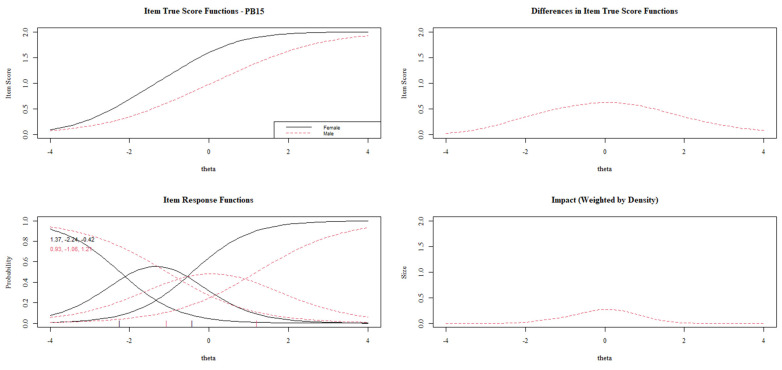
DIF plots for item PB15.

Figure 3, Figure 4, Figure 5, Figure 6 and Figure 7 show the diagnosis plots for each item with DIF. While interpreting the plots, we should keep in mind that items PB1 to PB4 retain two response categories from the original 3-point Likert scale because the program, by default, retains those categories with a minimum of five cases per cell. Each figure displays four graphics: (1) the upper-left graphic presents the item characteristic curves (ICCs) for each group; (2) the upper-right plot shows the differences between the ICCs for each group; (3) the lower-left graphic shows item response functions for each group and the values for the slopes and category threshold values (in that order); (4) the lower-right plot shows the ICCs’ differences when weighted by the distribution for the focal group (males).

The visual analysis of item PB1 (Figure 3) indicates a DIF, especially on the lower levels of prosocial behavior, where females present higher item scores with the same theta value. Additionally, the item presents a non-uniform DIF, being more discriminant for males (2.11 vs. 1.01).

As can be seen in Figure 4, item PB4 only presents uniform DIF, which is more prevalent in the lower levels of prosocial behavior. As in item PB1, females tend to select higher categories than males with the same overall prosocial behavior. The graphics show the lack of large changes in the item slope between groups (1.38 vs. 1.83), which supports the null non-uniform DIF.

Figure 5 offers DIF results for item PB12. Although the DIF is mainly due to the first threshold (−1.9 vs. −2.9), as in previous items, item PB12 shows the opposite DIF. In this case, the males present a higher item score with the same level of overall prosocial behavior. Additionally, item PB12 shows a non-uniform DIF, with a higher slope value for females (2.37 vs. 1.46).

Figure 6 demonstrates that item PB13 presents a slightly uniform DIF, benefiting male results. Contrary to the previous items, in this case, the DIF is more pronounced in the middle levels of prosocial behavior.

**Figure 8:**
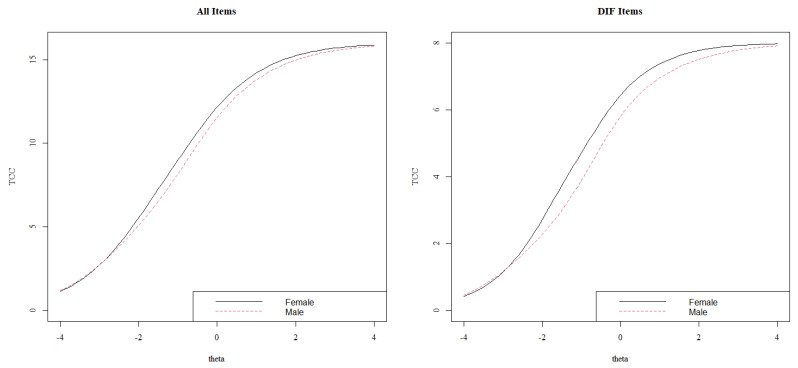
Tests characteristics curves (TCCs) by group.

Lastly, Figure 7 displays a uniform DIF for item PB15. As in most of the items with DIF, females present a higher item score than their male counterparts with the same overall prosocial behavior. Once the DIF has been analyzed item by item, Figure 8 shows the impact of these DIFs on the test characteristic curves (TCCs). The DIF effect on TCCs seems to be scarce while considering all the items, which is in line with the interpretations of the *pseudo*R^2^ differences.

**Figure 9:**
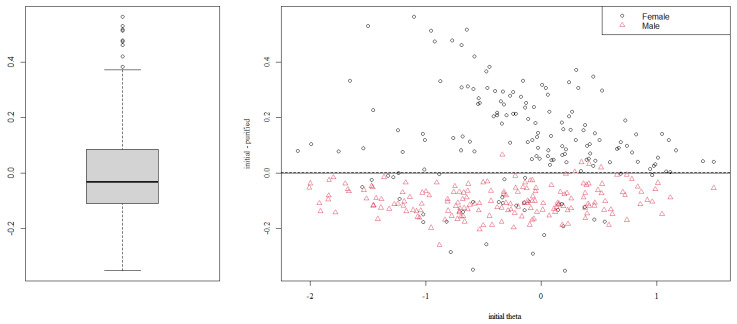
Individual-level DIF impact.

The analysis of the individual-level DIF impact complements the DIF exploration. The boxplot in Figure 9 indicates the differences between the individual scores ignoring DIF and those that consider it (purified). The median difference in theta estimation for the global sample is close to zero. However, the second plot in Figure 9 indicates that, in all levels of prosocial behavior, the females, in almost all cases, would receive a lower score if we account for DIF. Contrarily, the correction for DIF would lead to higher scores in males.

### 3.4. Nomological Evidences for Validity

The nomological validity of the scale was assessed by correlating the prosocial behavior (PB) results with other theoretically related constructs, such as the psychological capital subdimensions, namely, self-efficacy, hope, resilience, and optimism. Table 5 presents all the correlations. Additionally, an aggregated measure of the items without DIF (PB*) was calculated and included in the correlations. All the correlations were positive and statistically significant (*p* < 0.001). The correlation between the Prosocial Behavior Scale with and without items with DIF is positive and high (r = 0.89, *p* < 0.001). Contrasts between the validity coefficients obtained with PB and PB* show no statistically significant differences (*p* > 0.05), indicating a small or null impact of the DIF in the nomological validity of the scale.

## 4. Discussion

This paper aims to conduct the first psychometric study of the PBS in a Spanish sample. In doing so, we offer evidence of its one-dimensionality, reliability, differential item functioning, and nomological validity. Our results support the use of the scale in Spanish-speaking contexts as a brief and robust measure of global prosocial behavior. Additionally, we develop the study of the conceptualization of prosocial behavior by considering gender differences.

The unidimensional factor structure adequately fitted the data, and all the items presented factor loadings above 0.3, which is in line with the previous study that reported PBS factor structure in a similar sample [26]. Its reliability was also adequate and fit into the range of reliability results of previous studies [18,19,20,21,22,23,24,25,26,27,28,29,30,31,32].

Regarding the DIF study of PBS items, our results could be considered consistent with previous literature. Although half of the items presented DIF, its effect on the global scale functioning may be considered negligible. However, some insights arise from them. Firstly, the measurement of prosocial behavior using this scale benefits females’ scores. The DIF analysis could help us to understand those behaviors that are more characteristic in males and those that are more prevalent in females’ prosocial behavior. The items that did not present a DIF were the ones related to sharing behaviors, such as PB7 (“I share things I like with my friends”) or PB10 (“I let others use my games”), the helping behavior with classmates’ homework (PB9), and items PB2 and PB5, related to the time spent with friends and kindness, respectively. These results are similar to those reported by Eisenberg and Fabes [34].

Our results suggest that these kinds of prosocial behaviors would be equally present in females and males. Contrarily, differences emerged in the rest of the scale. Females presented a higher probability to manifest a higher score on those items related to emotional support as PB1 (“I try to make sad people happier”), PB4 (“I try to help others”), and PB15 (“I hug my friends”). This result agrees with Xiao et al. [38], who found that females present higher results on the emotional dimension of the PTM-R as well as in the results of the qualitative studies with adolescents [74]. The only items where males performed better were PB12 (“I like to play with others”) and PB13 (“I trust others”).

These results should not be interpreted as innate differences between men and women. For example, although some studies presented gender differences in empathy [75], these differences could not exist on natal levels of empathy [76]. Rather, they would be attributable to different patterns of socialization [77]. It would be the predominant culture that favors the appearance of some behaviors and not others in each gender [78]. Additionally, another factor that could artificially generate gender differences is the self-reported method, where females could tend to declare higher prosocial behavior to show congruency with the female stereotype of “kindness” [34,78,79]. All of these aspects should be kept in mind while developing prosocial behavior measures and when studying gender differences. Nielson et al. [37] developed a longer multidimensional scale that includes twenty items classified into five categories (defending, emotional helping, inclusion, physical helping, and sharing), trying to balance male characteristics behaviors with female ones. They did this to generate a scale that offers a comparable total score.

Finally, we tested the nomological validity of the scale by correlating the total prosocial behavior score with the subdimensions of the psychological capital. As expected, all the correlations were positive and statistically significant. Our results were similar to those found in an adult sample by Aydin and Aslan [80], who also found that the subdimension with the lowest relationship with prosocial behavior was resilience. Zhang et al. [63], through a longitudinal study, tested the predictive power of psychological capital on prosocial behavior, verifying its antecedent role. In this case, only optimism and self-efficacy showed statistically significant coefficients. Studies that included psychological capital as a global dimension also reported a positive effect on prosocial behavior [63,81]. Consequently, the PBS scores reproduce the relationships previously found in the literature with other prosocial behavior scales.

Our study presents some limitations. First of all, it is based on a cross-sectional sample of high school students from Spain. Consequently, the generalizability of the psychometric properties reported to primary students or Latin American countries requires further research. Future studies may propose longitudinal investigations to ensure scale invariance over time. These studies will make the results obtained from different educational levels comparable and will guarantee the robustness of the studies of the development of prosocial behavior during education. Additionally, the inclusion of more than one time measurement would allow future researchers to complement the psychometric study with evidence of test–retest reliability. As another limitation to be acknowledge, gender has been considered only as a dichotomous variable. Other variables, such as gender-role orientation, would be also interesting to understand regarding the interaction of the individual gender alignment with the DIF.

In summary, the PBS has shown adequate psychometric properties and, when the items are administered together, their gender bias is negligible. Consequently, it could be a good alternative for researchers or practitioners who need to administer a global and relatively short prosocial behavior scale. Additionally, our results indicate that a consideration of the specific characteristics of the prosocial behavior in each gender is crucial to properly understand gender differences without biases that systematically benefit females or, less frequently, males. Once we more accurately approach an understanding of these differences, we could better design more effective initiatives to promote sustainable prosociality.

## Figures and Tables

**Table 2:** Item content.

	Content
PBS1	I try to make sad people happier
PBS2	I spend time with my friends
PBS3 *	When I have to do things that I don’t like I get mad
PBS4	I try to help others
PBS5	I am gentle
PBS6 *	I cry about things thet don’t matter
PBS7	I share thins I like with my friends
PBS8 *	I feel annoyed
PBS9	I help others with their homework
PBS10	I let others use my toys
PBS11 *	I have bad dreams
PBS12	I like to play with others
PBS13	I trust others
PBS14 *	I bite my fingernails
PBS15	I hug my friends

Notes: * Control items.

**Table 3:** Means (M), standard deviations (SD), skewness (g1), kurtosis (g2), inter-item correlations, and corrected item-total correlations (*r_it_*).

	M	SD	g1	g2	PB1	PB2	PB4	PB5	PB7	PB9	PB10	PB12	PB13	*r_it_*
PB1	2.75	0.48	−1.68	1.93										0.44
PB2	2.70	0.50	−1.29	0.58	0.15 *									0.30
PB4	2.73	0.47	1.36	0.62	0.52 *	0.07ns								0.48
PB5	2.70	0.50	−1.33	0.73	0.29 *	0.09ns	0.39 *							0.41
PB7	2.50	0.60	−0.75	−0.41	0.18 *	0.19 *	0.19 *	0.15 *						0.42
PB9	2.24	0.63	−0.25	−0.65	0.28 *	0.05ns	0.34 *	0.21 *	0.21 *					0.37
PB10	2.40	0.65	−0.61	−0.61	0.24 *	0.14 *	0.27 *	0.19 *	0.28 *	0.24 *				0.42
PB12	2.66	0.57	−1.50	1.24	0.18 *	0.30 *	0.15 *	0.28 *	0.31 *	0.19 *	0.34 *			0.46
PB13	2.37	0.62	−0.45	−0.66	0.15 *	0.25 *	0.16 *	0.26 *	0.26 *	0.11 *	0.22 *	0.30 *		0.39
PB15	2.25	0.76	−0.46	−1.14	0.28 *	0.16 *	0.35 *	0.24 *	0.25 *	16 *	0.23 *	0.21 *	0.20 *	0.41

Notes: * *p* < 0.001; ns = non-statistically significant.

**Table 4:** Chi-square tests and changes in McFadden’s *pseudo*R^2^ for DIF detection.

	Model 1 vs. Model 2		Model 1 vs. Model 3		Model 2 vs. Model 3	
	*p*-Value χ^2^ Test	*Pseudo*R^2^ ∆ McFadden	*p*-Value χ^2^ Test	*Pseudo*R^2^ ∆ McFadden	*p*-Value χ^2^ Test	*Pseudo*R^2^ ∆ McFadden
PB1	0.000	0.053	0.000	0.088	0.000	0.035
PB2	0.082	0.006	0.199	0.006	0.645	0.000
PB4	0.000	0.045	0.000	0.053	0.054	0.007
PB5	0.847	0.000	0.968	0.000	0.867	0.000
PB7	0.921	0.000	0.403	0.002	0.179	0.002
PB9	0.038	0.005	0.053	0.007	0.212	0.002
PB10	0.190	0.002	0.290	0.003	0.383	0.000
PB12	0.000	0.025	0.000	0.038	0.004	0.013
PB13	0.000	0.016	0.002	0.016	0.630	0.000
PB15	0.000	0.084	0.000	0.087	0.106	0.003

**Table 5:** Nomological validity.

	Self-Efficacy	Hope	Resilience	Optimism	PB	PB *
Self-efficacy	1	0.382	0.322	0.361	0.295	0.256
Hope		1	0.292	0.504	0.309	0.309
Resilience			1	0.292	0.149	0.152
Optimism				1	0.261	0.245
PB					1	0.887
PB *						1

Notes: All the correlations are statistically significant *p* < 0.001; PB = Prosocial Behavior; PB * = Prosocial Behavior scale without items with Differential Item Functioning.

## Data Availability

The data presented in this study are available on request from the corresponding author.
